# Computer-Based Training in Math and Working Memory Improves Cognitive Skills and Academic Achievement in Primary School Children: Behavioral Results

**DOI:** 10.3389/fpsyg.2017.02327

**Published:** 2018-01-09

**Authors:** Noelia Sánchez-Pérez, Alejandro Castillo, José A. López-López, Violeta Pina, Jorge L. Puga, Guillermo Campoy, Carmen González-Salinas, Luis J. Fuentes

**Affiliations:** ^1^Department of Basic Psychology and Methodology, Faculty of Psychology, University of Murcia, Murcia, Spain; ^2^School of Social and Community Medicine, University of Bristol, Bristol, United Kingdom; ^3^Department of Health Sciences, Faculty of Health Sciences, Catholic University of Murcia, Murcia, Spain; ^4^Department of Developmental Psychology and Education, Faculty of Psychology, University of Murcia, Murcia, Spain

**Keywords:** working memory training, executive functions, mathematical achievement, reading ability, school children

## Abstract

Student academic achievement has been positively related to further development outcomes, such as the attainment of higher educational, employment, and socioeconomic aspirations. Among all the academic competences, mathematics has been identified as an essential skill in the field of international leadership as well as for those seeking positions in disciplines related to science, technology, and engineering. Given its positive consequences, studies have designed trainings to enhance children's mathematical skills. Additionally, the ability to regulate and control actions and cognitions, i.e., executive functions (EF), has been associated with school success, which has resulted in a strong effort to develop EF training programs to improve students' EF and academic achievement. The present study examined the efficacy of a school computer-based training composed of two components, namely, working memory and mathematics tasks. Among the advantages of using a computer-based training program is the ease with which it can be implemented in school settings and the ease by which the difficulty of the tasks can be adapted to fit the child's ability level. To test the effects of the training, children's cognitive skills (EF and IQ) and their school achievement (math and language grades and abilities) were evaluated. The results revealed a significant improvement in cognitive skills, such as non-verbal IQ and inhibition, and better school performance in math and reading among the children who participated in the training compared to those children who did not. Most of the improvements were related to training on WM tasks. These findings confirmed the efficacy of a computer-based training that combined WM and mathematics activities as part of the school routines based on the training's impact on children's academic competences and cognitive skills.

## Introduction

Student academic achievement plays a central role in future development outcomes, such as later achievements and educational aspirations (Marjoribanks, 2005), employment goals (Caspi et al., 1998), and socioeconomic success (Guglielmi, 2008). Among all academic competences, mathematics has been identified as an essential skill for international leadership as well as for disciplines related to science, technology, and engineering (Jordan et al., 2010). Given its positive consequences, researchers have designed training programs to improve children's mathematical skills (Starkey et al., 2004; Bryant et al., 2008; Dowker and Sigley, 2010; Ehlert and Fritz, 2013; Holmes and Dowker, 2013; Schacter and Jo, 2016). Moreover, executive functions (EF) have been related to positive school functioning (Clair-Thompson and Gathercole, 2006; Riggs et al., 2006; Brock et al., 2009; Razza and Blair, 2009; Best et al., 2011), which has resulted in a strong effort to develop EF training programs to improve students' skills and academic achievement (Wong, 2008; Thorell et al., 2009; Goldin et al., 2014; Söderqvist and Bergman-Nutley, 2015; Studer-Luethi et al., 2015; Traverso et al., 2015). Thus far, interventions have usually required trained professionals, changes in the scholastic curriculum, or lab-like environments. These characteristics decrease the possibility of effectively embedding the training tasks into the students' academic routines. Moreover, most of the training programs have exhibited near transfer effects, but they have often failed to prove far transfer effects that are highly related to school success, such as IQ or school grades (Melby-Lervag and Hulme, 2013; Sala and Gobet, 2017). Given the need to develop effective training programs and to clarify the effects of those programs by addressing previous limitations, we designed a computer-based training program composed of two components, namely, working memory and mathematics tasks, to be implemented by teachers during school hours. To assess the efficacy of the training, we considered the effects on a sample of school-age students' cognitive skills (EF and IQ), math and reading abilities as measured by standardized tests and school achievement as measured by math and language grades.

### Training programs

#### Executive functions training

Executive functions are defined as a set of general purpose mechanisms that regulate action and cognition (Miyake and Friedman, 2012). They are commonly composed of three related, albeit separate, components: *shifting*, which involves moving back and forth between multiple tasks, operations, or mental sets; *updating*, which requires monitoring and actively manipulating working memory representations; and *inhibition*, which is the ability to deliberately inhibit a dominant, automatic, or prepotent response (Monsell, 1996; Miyake et al., 2000; Clair-Thompson and Gathercole, 2006; Miyake and Friedman, 2012). These cognitive skills have been associated positively with several academic and socioemotional outcomes, such as mathematical achievement, adaptive and learning-related behaviors, and social competences (Clair-Thompson and Gathercole, 2006; Riggs et al., 2006; Brock et al., 2009; Razza and Blair, 2009; Best et al., 2011).

During their school years, children must deal with academic and social challenges that require them to successfully implement EF. For instance, students must switch quickly from one subject to another and transition from one academic task to another and from one skill to another in response to teachers' instructions. They must also remember and manipulate academic information and drop irrelevant data and add new data to update their skills. Moreover, children are required to inhibit dominant, automatic responses, such as being distracted by a classmate, and instead, remain focused on the teacher. These EF skills allow children to self-regulate their behavior and their academic performance.

Given the essential role of EF in children's successful development, numerous training programs have been developed to improve children's EF skills. For instance, the Tools of the Mind curriculum (Bodrova and Leong, 2007) is based on activities embedded in the school curricula, such as tasks to help self-regulate private speech and dramatic role playing and facilitate memory and attention. Diamond et al. (2007) applied this approach with 4- and 5-year-old children who received the training for 1 year, during which time the teachers spent approximately 80% of each day promoting EF skills. Another example is the play-based approach, such as the intervention developed by Traverso et al. (2015), in which children act out roles and have to collaborate to reach specified goals (30 min, 3 times per week for 1 month). In both cases, the participants of the training were preschoolers, and the results indicated improved EF skills (Diamond et al., 2007; Traverso et al., 2015). As a disadvantage, however, implementing these types of programs required psychologists and trained teachers to introduce methodological changes to the academic curricula. In addition, the implementation of these programs in primary schools may depend largely on specific reforms implemented by education policy makers.

#### Mathematics school-based interventions

Apart from the EF trainings, mathematics interventions have also been related to student academic improvements (Starkey et al., 2004; Bryant et al., 2008; Ehlert and Fritz, 2013). For example, Bryant et al. (2008) developed a program in which trained tutors enhanced children's mathematical skills by having the children work in small groups where they incorporated strategies such as modeling, thinking aloud, guided practice, and error correction. Their results revealed significant improvements in children's math skills and achievement levels. In the case of Starkey et al. (2004), children's math knowledge was enhanced through the implementation of three training strategies: classroom activities incorporated by the teachers into the math curriculum; teacher trainings designed to increase their understanding of children's mathematical development and enable them to implement the intervention; and mathematics classes administered at home that involved parents and children. Even with significant improvements, these types of training require teachers to prepare general tasks, and thus, they do not customize teaching to fit the level of each student. In contrast, computerized activities allow every child to progress at his/her own pace. Accordingly, a computerized mathematics game can be incorporated into the individual student's routine and adapted to the child's specific level of performance.

### Children's characteristics related to academic performance

When assessing the effectivity of any training program, it is necessary to take into account a set of children's characteristics that have been associated with student academic achievement. Specifically, in this study, we considered three of the most researched variables as our control variables: temperament, socioeconomic status (SES), and gender.

Regarding children's temperament, effortful control (EC) and negative emotionality (NE) have been extensively related to academic achievement (Gumora and Arsenio, 2002; Valiente et al., 2007, 2010, 2011, 2012, 2013; Neuenschwander et al., 2012). EC involves the individual differences in the self-regulatory process, such as attention, inhibition control, and activation control, whereas NE refers to children's negative reactivity and includes emotions such as anger, sadness, discomfort, fear, and shyness. Previous findings have revealed that students' EC contributes positively to academic performance (Neuenschwander et al., 2012; Valiente et al., 2013). In contrast, children's NE is negatively associated with school achievement (Gumora and Arsenio, 2002; Valiente et al., 2010, 2012). With respect to SES, there is vast literature showing the impact of family SES on student academic achievement and indicating that low-family income is related negatively to children's academic success (Hart and Risley, 1995; McLoyd, 1998; Davis-Kean, 2005; Valiente et al., 2011; Carvalho and Novo, 2012; Hoff, 2013; Sánchez-Pérez et al., submitted; for a meta-analytic review, see Sirin, 2005). Finally, gender has yielded inconsistent results across studies (see Davis-Kean, 2005; Valiente et al., 2007; Neuenschwander et al., 2012; Sánchez-Pérez et al., 2015, for contrasting results). Therefore, we considered students' gender, temperament (i.e., EC and NE) and SES as potential control variables.

### The present study

The motivation for the present study began with the requirement of some schools to improve the academic achievement of their students, mainly mathematical skills. Given this need, and the aforementioned relevance of some cognitive abilities for students to success at school, our research group designed a computer-based training program aimed to improve children's cognitive skills and school achievement, in a sample of typically developing school-age children.

Thorell et al. (2009) suggested that “as cognitive functions may vary in how easily they can be improved through training; focusing on specific cognitive functions and thereafter possibly use a combination of those training paradigms that have documented effects, appears to be the most rational approach” (Thorell et al., 2009, p. 107). Following that recommendation, we included two components in our training program. One involved computerized WM tasks due to their significant improvements on children's cognitive skills and school achievement. Moreover, because students' math skills were the main concern of the school management team, a commercial software product that teaches and reinforces mathematical skills was introduced as the second training component. Finally, with the aim to keep students engaged in the training tasks, an external reward system was also introduced. These activities were designed to be implemented by teachers as part of the daily school activities.

A wide range of potential training effects were taken into consideration to assess the impact of the training program as a whole, but also the effects of each component of the training program (WM and math exercises, respectively) on children's performance. On the basis of previous findings, we hypothesized that our training program will produce improvement in children's cognitive skills such as EF and intelligence (IQ); and in school achievement, mainly math and reading skills.

#### Improvement in cognitive skills

There is evidence that children who performed WM, planning, and inhibitory control computerized games had positive near transfer effects similar to those of trained skills and had a positive impact on students' school grades (Goldin et al., 2014). Interventions focused on working memory (WM) have also shown near transfer effects, such as improvements in visual (Thorell et al., 2009; Wong et al., 2014; Studer-Luethi et al., 2015) and verbal WM (Thorell et al., 2009; Wong et al., 2014) skills. Given previous findings and the well-established co-occurrence of WM and the other executive functions, namely, shifting and inhibition, we hypothesized that WM training will produce improvement in the other two EF components.

Working memory is also positively associated with intelligence. The association seems to be mediated by high-level attentional control involving the prefrontal cortex (Conway et al., 2003). Attentional control is needed to actively maintain task-relevant information in the presence of internal and external sources of distraction (Unsworth et al., 2014). A recent study has shown that training the updating component of WM through the *n*-back task increased participants' IQ (Jaeggi et al., 2008). Accordingly, we hypothesized that WM training will increase children's intelligence.

#### Improvement in academic achievement

Concerning academic achievement, WM training has been associated with improvement in math grades (Holmes and Gathercole, 2014), math standardized tests (Söderqvist and Bergman-Nutley, 2015), and arithmetic abilities (Bergman-Nutley and Klingberg, 2014). WM skills are likely to be required to master math competences, such as counting, mental arithmetic, measurement abilities, and space abilities (for a meta-analysis review, see Friso-van den Bos et al., 2013). Consequently, we expected to find a significant increment in mathematical skills in those children who practiced the WM activities.

Working memory training has also be shown to increase reading skills (Loosli et al., 2012; Karbach et al., 2015; Söderqvist and Bergman-Nutley, 2015) and vocabulary (Studer-Luethi et al., 2015), possibly due to the observed correlation between some reading aspects (e.g., reading comprehension and spelling) and verbal WM (Seigneuric et al., 2000; Pham and Hasson, 2014). Reading improvement may be due to the phonological storage component of WM, which has been shown to be a relevant factor for the development of a variety of linguistic abilities such as reading, vocabulary, and comprehension (Gathercole and Baddeley, 1990). Accordingly, we hypothesized that our training program would boost several aspects of children's reading skills.

#### The current computer-based training program

Our computer-based training program presents several advantages not found in most of the aforementioned interventions. First, the ease with which it can be implemented in school settings and the ease by which the difficulty of the tasks can be adapted to fit the child's ability level. Second, most of previously mentioned interventions have been conducted in lab-like environments, where it appears that the laboratory was moved to a school context. In this sense, the training may be perceived as a supplemental, short-term activity implemented in the schools, and as such, it fails to garner teacher commitment. Our program was designed to be integrated into the school routine based on the characteristics of the school environment; thus, it did not involve moving the lab environment into the school context. The training program was conducted by designated teachers who had undergone a short training program. Third, the tasks were designed to cover not just a specific grade, but rather several primary education grades. Moreover, because the students worked independently, the difficulty of the activities could be adapted to each child's ability and rhythm. Fourth, in the specific case of computer-based training, it is required that children perform repetitive tasks during mid-length to long interventions. Under such circumstances, the role of the child's engagement is crucial for the training program to achieve the highest level of efficacy. Previous researchers have found that external rewards increase intrinsic motivation (Cameron et al., 2005) and therefore may encourage children to continue performing repetitive tasks and, consequently, gain the potential benefits. We addressed the key point of the students' adherence to the training by including an engagement program.

## Materials and methods

### Participants

This study was performed on a final sample of 104 children (56 boys) aged from 7 to 12 years old (*M* = 9.17, *S.D*. = 1.20) who were enrolled in two schools from rural areas in the Region de Murcia (Spain). The control group included 53 children (29 boys; *M* = 9.26, *S.D*. = 1.27), and the training group was composed of 51 children (27 boys; *M* = 9.08, *S.D*. = 1.30). Informed consent forms were sent to 172 parents of children in grades 3–6. After two reminders, we obtained 137 participants. Children with special educative needs (18), those who were bilingual (9), and those whose families dropped out of the study (5) were excluded from the study, thus reducing the sample to 104 children.

### Measures

#### Pretest and posttest tasks

##### Cognitive skills

*Intelligence (IQ)*. We assessed children's IQ using the Spanish version of the Kaufman Brief Intelligence Test (K-Bit; Kaufman and Kaufman, 1990). This measure offers an index of verbal intelligence that covers knowledge of the language, the formation of verbal concepts and wealth of information and another index for non-verbal IQ, which requires non-verbal reasoning and flexible problem-solving skills. Cronbach's alpha ranged from 0.76 to 0.90 (verbal IQ) and from 0.77 to 0.93 (non-verbal IQ).

*Verbal working memory (digit span task)*. This task was based on the subtest of the Wechsler Intelligence Scale for Children (WISC-III, Wechsler, 2003). We used both the forward digit recall task, which is a measure of verbal short-term memory, and the backward digit recall task, which is a measure of verbal working memory. In the former, the child is told to repeat the digits in the same order as they were presented, whereas in the latter the child is required to recall a sequence of spoken digits in the reverse order. The stimuli were recorded by a woman's voice and presented aurally through the computer (see also Alloway et al., 2010).

*Shifting (dots task)*. The dots task has two conditions, congruent and incongruent (Davidson et al., 2006). The stimulus is a red heart or a flower, which will appear on the right or left of the screen. In the congruent condition, one rule applied, i.e., press on the same side as the heart. The incongruent condition also demands remembering a rule, i.e., press on the side opposite the flower, plus it requires inhibition of the tendency to respond on the side where the stimulus appears. The difference between these two conditions can be regarded as an indicator of the child's ability to task shift.

*Inhibition (go/nogo task)*. This task was adapted from Durston et al. (2002). Participants had to press a button on the joystick when a target animal (go trials) randomly extracted from a pool of 10 appeared and avoid responding when a particular non-target animal (a lion) appeared (no-go trials). Stimuli were presented for 500 ms. A variable interval ranging from 100 to 300 ms after responding or after 1100 ms had elapsed defined a new trial. The non-target could be presented after 1 (type 1), 3 (type 2), or 5 (type 3) go trials. The task started with a block of 10 practice trials, followed by 40 go trials. The participants completed an experimental block of 168 go/no-go trials. Thus, there were 14 no-go trials for each number of preceding go sequences, where the total number of go trials varied for the sequenced 1, 3 and 5 preceding go trials, i.e., 14, 42, and 70 go trials, respectively.

##### Academic achievement

*Mathematical ability*. Children's math abilities were assessed using the Spanish version of the Woodcock-Johnson III (WJ-III) Achievement battery (Woodcock et al., 2001), which had been validated for use with participants from age 6 to 13 years in Spain (Diamantopoulou et al., 2012). The mathematical abilities measured by this battery of tests include calculations, math fluency, applied problems and quantitative concepts. The subtest on calculations assesses the student's ability to perform simple mathematical computations, including addition, subtraction, multiplication, and division. Math fluency measures the ability to quickly solve simple calculations. Applied problems assesses the ability to analyze and solve math problems. Quantitative concepts measures the student's knowledge of mathematical concepts, symbols, and vocabulary. Apart from these individual scales, we also considered the W composite scores that WJ-III provided. These include broad math, which includes calculations, math fluency, and applied problems; brief math, which includes calculations and applied problems; math calculation skills, which includes calculations and math fluency; and math reasoning, which includes applied problems and quantitative concepts. The raw scores of each of these subscales were transformed into W scores (Woodcock and Dahl, 1971; Woodcock, 1978), according to the Rasch measurement model (Rasch, 1960; Wright and Stone, 1979).

*Reading ability*. Reading ability was assessed using five subtests, namely, name or sound of letters, same or different, word and pseudo-word reading, and punctuation marks, from the PROLEC-R (Batería de Evaluación de los Procesos Lectores — Revisada (Reading Process Evaluation Battery-Revised; Cuetos et al., 2007). On the name or sound of letters subtest, the child is asked to name or say the sound of 20 written letters. The same-different subtest analyzes whether the child can segment/identify the letters and whether s/he exhibits logographic reading. In this subtest, the child is presented with 20 pairs of stimuli (words or pseudo-words), and s/he is asked to report whether the two words or pseudo-words are the same or different (half of the 20 pairs were the same, and the other half were different). The word reading subtest measures the process of letter recognition by asking the child to read 40 words, 20 of which are high frequency appearing words and 20 of which are low frequency appearing words. The pseudo-word reading subtest evaluates the accuracy in identifying pseudo-words or non-existing words and indicates the ability to pronounce new or unknown words. As with the word reading subtest, the child is presented with a total of 40 stimuli to read. On the punctuation marks subtest, the child is asked to read a text attending to 11 punctuation marks, i.e., dots, commas, question, and exclamation marks. This subtest reflects the prosodic elements or intonation of the written language, such as the ability to separate or emphasize specific words in a sentence. All subtests have a score based on response accuracy (each correct answer adds one point) and a score based on the time required to complete the subtest (measured in seconds). Higher accuracy scores and shorter times to complete the tasks indicate better performance. To calculate an ability index for each subtest, the PROLEC-R provides the following equation: correct answers divided by time and multiplied by 100. As our interest is focused on overall reading achievement, we used a composite score formed by standardizing the five ability indexes and averaging the scores.

*Math and language grades*. Teachers reported children's grades in math and language subjects following the official five-mark system ranging from unsatisfactory (0) to outstanding (4).

#### Control variables

##### Temperament

Parents evaluated children's temperament using a Spanish version of the Temperament in the Middle Childhood Questionnaire (TMCQ; Simonds and Rothbart, 2006). Parents had to report the extent to which each statement properly described his/her child's behavior within the previous 6 months on a Likert-type scale ranging from 1 (almost always untrue) to 5 (almost always true), with an additional option of not applicable. The TMCQ assesses 4 higher-order dimensions of temperament. For the purpose of this study, we selected the items included in EC, defined as the ability to inhibit a dominant response so as to perform a subdominant response, to detect errors and to engage in planning (Rothbart and Rueda, 2005; Rothbart and Bates, 2006), and NE, which measures negative emotions such as anger, discomfort, fear, sadness, shyness and soothability (reversed). Cronbach's alphas were 0.86 and 0.93 for each dimension, respectively.

##### SES

Parents completed a questionnaire about father's and mother's years of schooling and their monthly family income ranging from 1 (*less than 750 Euros*) to 6 (*more than 3000 Euros*). An index of socioeconomic status was calculated by standardizing and averaging the three variables.

##### Gender

Child's gender was coded as (0) for girls and (1) for boys.

#### Training program

The training session started with the child inside a spaceship and four planets/satellites in front of him/her. Each planet/satellite represented a training task: The Fire planet represented the *n-*back task, the Earth denoted math activities, the Moon was the working memory span task, and the Ice planet signified the abstract shapes task. The tasks included on the Fire planet, the Moon, and the Ice planet formed the WM training created by our research group, whereas the activities on the Earth conformed to the math training that was developed by the company Educamigos S.L.

The three tasks were included in the WM training (for more details, see the Supplementary Material). The *n*-back task, which was based on Pelegrina et al. (2015), required children to pay attention to a sequence of items and then determine whether a stimulus presented on the screen matched a stimulus previously presented. The set of stimuli involved shapes, drawings and words, such as vehicles, fruits, and animals, or alphanumeric stimuli, such as digits and letters. In the abstract shapes task, which was based on Davidson et al. (2006), children were taught a rule for each stimulus, i.e., press the right button for this stimulus and press the left button for that stimulus. The number of rules and stimuli were increased, and the children had to remember all of this. In this task, geometric shapes comprised the set of stimuli. The third exercise was a working memory span task, based on (Petrides, 1995), in which children were required to select the stimulus that had not been presented in the previous set of stimuli, including animals, fruits, shapes, letters, vehicles, cartoons, toys, and non-common animals.

The math training (developed by Educamigos S.L.) consisted of a set of exercises that increased in complexity that allowed students to practice basic math skills, such as addition, subtraction, division, multiplication, and mental arithmetic (see Supplementary Table 1 for further details).

With the aim to engage children in WM and math training, students from the training group were involved in a bonus system in which they received scores (called *floros*) based on their performance on training activities. At the end of the posttest, children exchanged their *floros* for a reward (e.g., a book, a ball). Rewards were grouped into three categories, i.e., high, medium, and low, based on the number of *floros*. The more *floros* a student had, the better the reward s/he could choose.

### Procedure

The study protocols were approved by the Ethics Committee of the University of Murcia, and the study was performed in accordance with the approved guidelines and the Declaration of Helsinki, with written informed consent from all participants. To participate in the study, informed consent of the parents was required, and hence, informed consent forms were sent to the families. Once consent was obtained, questionnaires regarding their children's temperament and socioeconomic information were delivered to the parents, and the parents were requested to complete and return the questionnaires to the school.

This study followed a longitudinal design with three phases, namely, pre-training, training, and post-training, and both groups were required to complete pre- and post-assessments. The pretest took approximately 3 weeks and was then followed by 13 weeks of training for the experimental group. One week following the end of the training, children from both groups were reassessed. Computer and written tasks were divided into five sessions and were administered by trained assistants. In three sessions, which were individually administered, the children were asked to reply in written or oral form to pencil and paper tests (tests of math and language skills and the K-Bit) and in oral form to some computer tasks (e.g., digit span). The remaining computer tasks were administered to groups of students of the same age with a maximum of 12 children per group. Assistants explained the instructions, and one assistant was assigned to every two children to verify that the children understood the tasks and were completing the tasks appropriately. Assistants were counterbalanced between sessions such that no child was ever evaluated twice by the same assistant. All tasks followed a counterbalanced sequence to avoid systematic variations arising from the order of administration. Random ordering protocols with a table of random digits for each child to achieve counterbalance were used, and oral consent from participants were obtained prior to testing sessions. Teachers reported children's math and language grades before and after the intervention.

The training phase included two weekly 30-min sessions over 13 weeks. For the children in the experimental group, during the first part of each session, they completed math training exercises, and in the second part, they engaged in WM tasks. The training programs were administered in the computer classroom where each child completed the tasks on a computer following the instructions of a trained teacher. While the experimental group was involved in training activities, the active control group from the other school engaged in the standard activities usually programmed for the children as they interacted with computers in the computer classroom. These activities trained the child to use computers through a variety of tasks; however, there was no connection to EF. The control group sessions were of the same duration as the intervention sessions.

### Statistical analyses

First, we tested potential sociodemographic and temperamental differences between control and training groups at baseline by running independent *t*-tests. Furthermore, age and gender effects were verified using analyses of variance (ANOVAs) and independent *t*-tests, respectively. Next, we examined whether the training group outperformed the control group in EF and academic measures by comparing the scores between the pretest and the posttest for the two groups using analyses of covariance (ANCOVAs). Finally, general linear model analyses were run to analyze the contribution of each training program to the improvements realized by the experimental group.

## Results

### Differences between training and control groups at baseline

As presented in Table 1, no significant differences between the control and the training groups were found at baseline with respect to mothers' and fathers' ages, children's mean age and children's EC. Thus, as NE and family SES yielded significant differences between both groups, we considered them as covariates in further analyses.

**Table 1:** Training and control groups' means, standard deviations and comparison of families' socio-demographic characteristics and children's temperament at baseline.

	**Training group**	**Control group**		
Mother's age	39.00 [5.10]	39.43 [6.24]	*t*_(96)_ = 0.37, *p* = 0.710	No difference
Father's age	41.80 [5.25]	41.53 [6.03]	*t*_(88)_ = −0.22, *p* = 0.823	No difference
Family's SES	0.21 [0.76]	−0.22 [0.77]	*t*_(100)_ = −0.2.87, *p* = 0.005	Difference
Children's age	9.08 [1.13]	9.26 [1.27]	*t*_(102)_ = 0.79, *p* = 0.434	No difference
Children's EC	0.13 [2.75]	−0.14 [1.98]	*t*_(93)_ = −0.53, *p* = 0.588	No difference
Children's NE	−0.83 [4.47]	0.97 [4.15]	*t*_(93)_ = 2.02, *p* = 0.046	Difference

Independent *t*-tests revealed significant gender effects for the go/nogo and dots variables. To examine the age effect, we considered the child's grade for the analyses. In this case, ANOVA yielded significant differences for go/nogo, dots, IQ, grades and scores on math and language standardized tests. Therefore, children's grades and gender were also included as covariates in the subsequent analyses.

### Differences between training and control groups at posttest

To test the efficacy of the training, we conducted ANCOVAs to compare performances between the training and control groups using pretest scores for each task as covariates. The results revealed a significant effect of group on posttest scores, after controlling for the child's pretest scores, gender, grade, EN and SES, on the following tasks (see Table 2): non-verbal IQ *F*_(1, 86)_ = 4.77, *p* = 0.032, $\eta_{p}^{2}$ = 0.05; percentage of errors on go/nogo task type 1 *F*_(1, 81)_ = 4.84, *p* = 0.030, $\eta_{p}^{2}$ = 0.06, type 2 *F*_(1, 81)_ = 8.85, *p* = 0.004, $\eta_{p}^{2}$ = 0.10, type 3 *F*_(1, 81)_ = 10.07, *p* = 0.002, $\eta_{p}^{2}$ = 0.11, total nogo, *F*_(1, 81)_ = 12.80, *p* = 0.001, $\eta_{p}^{2}$ = 0.14; math fluency *F*_(1, 86)_ = 6.78, *p* = 0.011, $\eta_{p}^{2}$ = 0.07, math grade *F*_(1, 85)_ = 19.80, *p* < 0.000, $\eta_{p}^{2}$ = 0.19; and reading ability *F*_(1, 86)_ = 9.76, *p* = 0.002, $\eta_{p}^{2}$ = 0.10. For all of these tasks, the results indicate that children who participated in the training performed better than children who did not, i.e., the control group.

**Table 2:** Training vs. control groups on the pre- and post-training assessments: means, standard deviations, the results of ANCOVAs (control vs. training groups) and effect sizes (partial eta squared).

**Task**	**Group**	**Pre-training**		**Post-training**		**Group effect**		
		**Mean**	***SD***	**Mean**	***SD***	***F***	**Direction**	**Effect size**
**WJ-III SCORES**								
Calculation	Control	15.68	3.50	17.93	2.80	1.21	No difference	0.01
	Training	17.16	2.75	19.02	2.77			
Math fluency	Control	52.60	20.87	59.88	20.80	6.68^*^	Training > control	0.07
	Training	47.69	14.63	61.10	15.55			
Applied problems	Control	33.06	5.12	35.62	5.80	0.09	No difference	0.00
	Training	35.51	4.74	37.59	4.04			
Quantitative concepts	Control	31.19	5.74	34.05	5.30	0.30	No difference	0.00
	Training	33.53	4.33	35.33	3.76			
Broad math	Control	493.62	12.05	500.36	10.95	0.70	No difference	0.01
	Training	497.78	8.87	504.20	8.40			
Brief math	Control	492.79	14.88	501.81	13.53	0.39	No difference	0.00
	Training	499.88	11.50	507.25	12.28			
Math calculation skills	Control	495.85	10.16	501.71	7.84	0.82	No difference	0.01
	Training	497.78	7.45	503.88	7.55			
Math reasoning	Control	492.71	17.52	501.67	17.58	0.12	No difference	0.00
	Training	501.04	13.11	507.45	11.83			
**PROLEC**								
Reading abilities	Control	0.05	0.96	−0.04	0.89	9.76^**^	Training > control	0.10
	Training	−0.05	0.72	0.14	0.77			
**SCHOOL GRADES**								
Math grades	Control	2.36	1.43	1.85	1.44	19.80^***^	Training > control	0.19
	Training	2.86	0.89	3	0.92			
Language grades	Control	2.40	1.44	2.37	1.45	0.30	No difference	0.00
	Training	2.96	0.92	2.82	1.03			
**K-BIT**								
Verbal IQ	Control	95.83	11.68	99.17	11.99	0.15	No difference	0.00
	Training	106.25	14	107.10	12.19			
Non-verbal IQ	Control	96.53	12.14	98.14	13.29	4.77^*^	Training > control	0.05
	Training	100.06	12.81	104.39	11.03			
**DIGIT SPAN**								
Forward recall	Control	4.40	0.77	4.62	0.66	0.03	No difference	0.00
	Training	4.61	0.78	4.80	0.80			
Backward recall	Control	3.06	0.80	3.40	0.83	0.78	No difference	0.01
	Training	3.33	0.55	3.41	0.73			
**DOTS**								
Shifting	Control	88.56	59.61	92.65	85.86	0.05	No difference	0.00
	Training	97.30	57.99	74.81	55.75			
**Go/NoGo (% ERRORS)**								
Type 1	Control	31.61	18.71	34.75	17.27	4.84^*^	Training > control	0.06
	Training	36.83	20.37	24.09	17.32			
Type 2	Control	32.37	16.97	34.36	19.77	8.85^**^	Training > control	0.10
	Training	30.81	20.28	20.59	14.68			
Type 3	Control	33.28	18.86	29.92	15.78	10.07^**^	Training > control	0.11
	Training	28.57	19.27	18.77	14.84			
Go	Control	3.55	4.17	2.42	3.25	0.00	No difference	0.00
	Training	6.40	15.04	2.65	10.36			
Nogo	Control	32.42	13.99	31.38	13.81	12.80^**^	Training > control	0.14
	Training	32.59	18.21	19.23	12.71			
Go (RT)	Control	437.13	56.82	427.56	51.59	0.14	No difference	0.00
	Training	433.19	41.81	429.00	56.26			

Asterisks denote the statistical significance level (

* p < 0.05;

** p < 0.01;

*** *p < 0.001)*.

### Specific contribution of training on children's improvement

Once we confirmed the efficacy of the global training program on children's cognitive skills and academic achievement, the next step was to test the contribution of each specific component of the training (WM and math) on those gains. First, an index of the child's performance for each component was calculated. To do this, we divided the attained level on each task by the number of sessions. In the case of the WM program, the child's performance on the *n*-back, abstract shapes, and WM span assessments were standardized and averaged to create a general WM performance. We combined these scores because they were significantly and positively correlated and achieved the following results: *n*-back and abstract shapes *r* = 0.49, *p* < 0.001; *n*-back and WM span *r* = 0.40, *p* = 0.004; abstract shapes and WM span *r* = 0.36, *p* = 0.009. Second, multiple linear regressions were computed using the posttest scores as the dependent variable, while in the case of the independent variables, the pretest scores, gender and course were introduced in the first step, and the child's performance on each component was introduced in the second step.

The results revealed a significant contribution of the WM component, considering pretest scores, gender and grade, to children's improvement in the following areas: non-verbal IQ, *F*_(5, 45)_ = 6.20, *p* < 0.001, *R*^2^_*adj*_ = 0.34; $\hat{\beta }=$ 0.27, *p* = 0.034, percentage of errors in go/nogo task type 2, *F*_(5, 45)_ = 1.45, *p* = 0.196, *R*^2^_*adj*_ = 0.05; $\hat{\beta }$ = −0.33, *p* = 0.025 type 3, *F* [5, 45] = 4.79, *p* = 0.001, *R*^2^_*adj*_ = 0.28; $\hat{\beta }$ = −0.46, *p* = 0.001, total nogo, *F* [5, 45] = 3.01, *p* = 0.020 *R*^2^_*adj*_ = 0.17; $\hat{\beta }=$ −0.39, *p* = 0.005; reading abilities, albeit this result was only marginally significant, *F*_(5, 45)_ = 30.42, *p* < 0.001, *R*^2^_*adj*_ = 0.75; $\hat{\beta }=$ 0.15, *p* = 0.074. In contrast, no one of the training components by itself can explain the children's improvement on the percentage of errors on the go/nogo task (type 1), math grades or math fluency. Increments in adjusted R-squared and lower and upper confidence intervals for the previous statistical analyses are shown in the Supplementary Material (Supplementary Tables 2, 3). Statistical analyses were also conducted including *floros* performance as an additional independent variable. Because the results did not vary, the results of these analyses are not reported.

## Discussion

The results indicated that students involved in the training group outperformed those in the control group in math fluency, math grades, reading abilities, inhibition, and non-verbal IQ. Moreover, most of these improvements were associated with their performance on WM tasks, suggesting that the WM intervention leads to more near and far transfer effects than the mathematical activities alone, although the contribution of both types of intervention must be considered to improve certain mathematical skills, i.e., math grades and math fluency.

### Training effects on cognitive skills

Our training program has exhibited positive and significant transfer effects on important cognitive skills related to school performance. As expected, our findings also indicated that the training group showed significantly better post-training performance on the inhibition measure (go/nogo task). Inhibition skills are essential for students to deal successfully with academic challenges. As Diamond et al. (2007) argued, this ability allows children to resist a preponderant irrelevant response and fosters the ability to focus and sustain their attention on academic tasks and goals, e.g., avoid being distracted by a classmate and instead focus attention on the teacher. Regarding the inhibition results, we found a pattern of improvement across all nogo types (1, 2, 3, and total). Improvements in inhibition have been found after WM training with clinical samples, i.e., ADHD children and young people with social, emotional and behavioral difficulties (Klingberg et al., 2005; Roughan and Hadwin, 2011). This far transfer effect could be explained by the well-established co-occurrence of WM and inhibition, which means that they support each other and are interdependent on one another (for a review, see Diamond, 2013). Thus, WM training sessions may help children to inhibit their prepotent responses by actively reminding them (WM) to follow the instructions.

Children who were involved in the training activities outperformed also those who were not involved in the training in non-verbal IQ. Even more, these gains were more related to the WM activities than to the math exercises. Previous WM training with ADHD children has also resulted in improvements in non-verbal IQ (Klingberg et al., 2002, 2005), although studies with typically developing children have not always yielded statistically significant improvement (Thorell et al., 2009; Bergman-Nutley et al., 2011; Loosli et al., 2012; Mansur-Alves et al., 2013; Studer-Luethi et al., 2015).

In explaining how the WM training yielded far transfer effects on children's non-verbal IQ, Conway et al. (2003) have suggested that WM capacity and IQ are highly related, being the basis of such relationship an executive-attention control mechanism that is mediated by the prefrontal cortex. Consistent with this notion, if children's WM skills are trained, it is expected that the connection with high-level cognitive skills would lead to an improvement in IQ.

### Training effects on academic achievement

**Figure 1:**
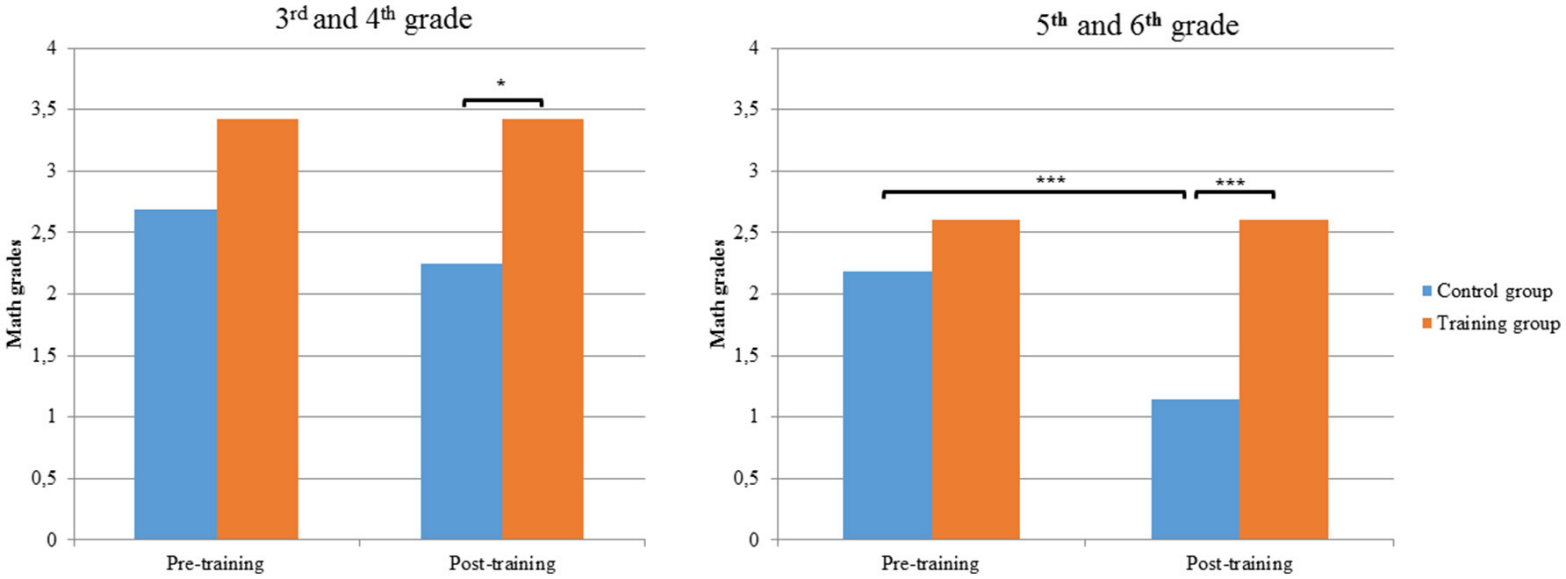
Training vs. control groups in math grades separated by stage: 2nd stage (3rd and 4th grade) and 3rd stage (5th and 6th grade); (^*^p < 0.05; ^***^p < 0.001).

Our training accomplished its primary target, i.e., to boost students' academic achievement, especially their math skills. Children who participated in the training received better math grades and exhibited greater increases in their math fluency and reading abilities compared with the children who did not participate in the training program. Surprisingly, with respect to math grades, we observed that final math scores in the control group were worse than those obtained at baseline. To explain this, teachers informed us that they tended to be stricter on math exams as the academic years progressed. Accordingly, math grades for those in the control group revealed a tendency whereby students received lower scores on the last math exam compared with the scores they obtained at the end of the previous academic year, which were treated as baseline scores (see Table 2). Importantly, such a negative tendency is more apparent in children in the 3rd stage (grades 5 and 6) than in children in the 2nd stage (grades 3 and 4) (see Figure 1). Thus, our training program not only appears prevent that negative tendency in math scores in the training group, but the training group children also improved their ability to solve calculations more quickly and efficiently (math fluency) than the non-training group of students. Previous findings have associated students' mathematical improvement with math interventions (Dowker, 2005; Bryant et al., 2008; Ehlert and Fritz, 2013) as well as with WM skills (Passolunghi et al., 2007; Peng et al., 2016). However, in the case of our children's math outcomes, gains were associated with performance on the whole training program and not related to either WM or math activities separately. This suggests that if we want our students to improve their mathematical abilities, we should train their WM skills in addition to providing educational reinforcement through extra math exercises.

In addition, children's reading abilities were better in the training group and, specifically, their gains were significantly associated with their WM performance rather than to math activities. These relations are consistent with previous studies, in which WM training related to improvements in children's language skills (Bryant et al., 2008; Ehlert and Fritz, 2013; Söderqvist and Bergman-Nutley, 2015; Studer-Luethi et al., 2015). The mechanism boosting these more efficient reading skills would be the influence that WM training has on the academic performance *via* the learning route. This route has been proposed by Bergman and Söderqvist (2017), throughout which WM capacity has a positive impact on academic outcomes by improving student learning capacity and attention. Furthermore, if children can focus better on reading activities and better assimilate new knowledge, they are more likely to master reading proficiency skills, such as decoding, metaphonological and prosodic skills earlier in life.

### Non-significant training effects

We did not find significant improvements in children's verbal WM, shifting, or language grades following the training. The non-significant improvement in verbal WM was unexpected based on previous studies (for a meta-analytic review, see Melby-Lervag and Hulme, 2013). This result might be explained by the task used to measure verbal WM. Students' scores on the digit span task, both forward and backward versions, did not vary widely, making it difficult to capture small changes in such skills when testing typically developing children. Regarding shifting, the lack of improvement in this component of the EF might be due to the scarce demands the training program tasks make on this ability. Training tasks focused mainly on keeping active contents in memory to update information in short-term memory (the *n*-back task), retain as many items as possible in memory (the span task), or keep active stimulus-response associations (the abstract shape task), abilities that do not require continuous attention nor task shifts.

Finally, language grades were reported by teachers who also reported on students' reading skills, an area where we found significant gains in the training group. However, as language scores usually integrate children's knowledge of Spanish grammar, literature, and syntax, among other academic competences, the learnings are probably more closely associated with students' learning-related behaviors rather than any specific cognitive training.

### Limitations and future directions

There are three major limitations in the present study to be considered. First, the control and the training groups were from two different schools, although they were chosen because they were in surrounding neighborhoods and the children shared similar educational environments. Second, our training is composed of two components, WM and mathematics, thus making it difficult to distinguish the specific effects of each training on student's improvement. Third, our post-training measures tested immediate effects, but they did not assess long-term effects. However, these limitations can be addressed in future research. It would be useful to analyze the specific effects of the two components of our training independently, in two different groups, and to follow up with the children over time to assess the long-term effects of the training.

## Conclusions

Our study confirmed the efficacy of a computer-based training program integrated into students' school routines that combined WM and mathematics activities to improve students' cognitive and academic skills. Specifically, compared with the control group, the training group exhibited significant gains on abilities highly related to children's academic success, such as math and reading abilities, non-verbal IQ, and inhibition skills, in a sample of typically developing school children. Today, our study contributes to the meta-analytic studies about the benefits of WM training in children (Melby-Lervag and Hulme, 2013; Sala and Gobet, 2017) by exploring the near and far effects of a computer-based WM and mathematics training program. These findings highlight that children's cognitive skills and academic achievement can be improved by a WM training program when training is integrated into school routines.

## Author contributions

LF conceived and designed the over-arching study. AC programmed and controlled the training software, CG-S and NS-P organized and interpreted the temperament questionnaire, the PROLEC-R, and the K-BIT test. NS-P and VP organized and interpreted the WJ III Mathematical Achievement battery. LF and GC organized and interpreted the executive functions tasks. NS-P, JL-L, and JP carried out and interpreted the statistical analyses. LF, NS-P, CG-S, and VP oversaw the testing and data collection. NS-P and LF drafted the manuscript. All authors approved the final version of the manuscript for submission.

### Conflict of interest statement

The authors declare that the research was conducted in the absence of any commercial or financial relationships that could be construed as a potential conflict of interest.
