# Supplementary material for: Computer-Based Training in Math and Working Memory Improves Cognitive Skills and Academic Achievement in Primary School Children: Behavioral Results
Source: Front Psychol. 2018 Jan 9;8:2327. doi: 10.3389/fpsyg.2017.02327 (PMC5767320; doi:10.3389/fpsyg.2017.02327)
Supplement: Supplementary file 1 [file DataSheet1.pdf]

Supplementary Material

Computer-based Training in Math and Working Memory Improves Cognitive Skills and Academic Achievement in Primary School Children: Behavioral Results

Noelia Sánchez-Pérez[1], Alejandro Castillo[1], José A. López-López[2], Violeta Pina[1], Jorge L. Puga[3], Guillermo Campoy[1], Carmen González-Salinas[4], and Luis J. Fuentes[1*]

*** Correspondence:** Luis J. Fuentes: lfuentes@um.es

1 Supplementary Information

Description of each cognitive training task used in this study.

1.1. *n*-back task

We manipulated a total of three variables, namely, memory load, presentation time, and stimuli type, to create multiple difficulty levels. Load WM was modified by using three levels of complexity (1-, 2-, 3-back). The presentation time was 500 ms (easy trials) or 1000 ms (difficult trials). Moreover, we had two difficult levels within each stimuli type: alphanumeric stimuli (small set with 5 elements and big set with 10 elements); shapes (simple and complex shapes, such as squares and drawings without key patterns, respectively); words (easy level with different semantic categories and difficult level with identical semantic categories), and drawings that the same levels as "words" stimuli).
The Supplementary Figure 1 displays the experimental design for the n-back task. Instructions were presented on the screen until the child was ready, i.e., s/he had to press a bottom to continue, and a white screen with the word loading then appeared for three seconds. Each trial started with a white screen (500 or 1000 ms, randomly assigned), and the stimulus was then presented for 500 or 1000 ms, depending on the level of difficulty. The child then had up to 5000 ms, from the time the stimulus first appeared on the screen, to respond. Finally, the child was given an audio feedback, which lasted 500 ms, for each response—one sound for correct responses and another for incorrect responses. Each block was composed of 3 trials that consisted of stimuli not presented previously, and 10 trials that consisted of 6 stimuli not previously presented and 4 stimuli previously presented. This sequence was the same for all types of n-back tasks. A 95% correction rate on a block was considered a successful performance for that block, and when the child successfully completed two blocks, s/he was advanced to the next level.

1.2 Abstract shapes task

In this task, we manipulated two variables, set size and time response, to create multiple difficulty levels. We had 4 set sizes, from the easiest level to the most difficult: 2 stimuli, where the child had to press the right button for one stimulus and the left button for the other stimulus; 4 stimuli, where the

child had to press the right button for two stimuli and the left button for the other two stimuli; 6 stimuli, where the child had to press the right button for three stimuli and the left button for the other three stimuli. There was also a level with 8 stimuli, where the child had to press the right button for four stimuli and the left button for the other four stimuli, but no one reached this level.

Within each set size, there were 5 levels of difficulty that were determined by the maximum time the child had to respond. In the easiest level, the child had 10 seconds to respond. We considered the child's average reaction times on that first block and estimated the maximum response times for the other levels using the following mathematical formulae: Average + SD/2 (2nd difficulty level); Average (3rd difficulty level); Average – SD/2 (4th difficulty level); Average – SD (5th difficulty level).

The experimental design for this task is presented in Supplementary Figure 2. As in the n-back task, instructions were presented on the screen until the child was ready, i.e., s/he had to press a bottom to continue, and a white screen with the word loading appeared for 3 seconds. Each trial started with a white screen (500 or 1000 ms, randomly assigned), and the stimulus was then presented. The child was required to respond within a specific time period that was dependent of the level of difficulty. On the same screen, the child received an audio feedback, which lasted 500 ms, for each response—one sound for correct responses and another sound for incorrect responses. Each block was composed of 24 trials. A 95% correct rate on a block was considered a successful performance for that block, and when the child successfully completed two blocks, s/he was advanced to the next level.

1.3. Working memory span task

We manipulated 2 variables, i.e., set size and retention time, to create different levels of difficulty. With respect to set size, from the easiest level to the most difficult level, we had 3, 4, 5, and 6 stimuli presented, including the target. The retention time, from when the set presentation finished and a question mark appeared (see Supplementary Figure 3 for the experimental design) to when the child must respond, also had four levels of difficulty: 1000, 2000, 4000, and 8000 ms.

As in the other tasks, instructions were presented on the screen until the child was ready (s/he had to press a bottom to continue), and a white screen with the word "loading" appeared for 3 seconds. Each trial began with a white screen (500 or 1000 ms, randomly assigned) that preceded the presentation of the stimuli (500 ms), between which a white screen appeared (500 ms). The stimuli presentation, the time of which was dependent on the level of difficulty, ended with a question mark, followed by a screen in which two stimuli appeared to which the child was required to reply (2000 ms). Once the child responded, an audio feedback was presented (500 ms, one sound for correct and another for incorrect). Each block was composed of 20 trials. When the child achieved 95% accuracy in a block, it was considered a successful performance, and when the child successfully completed two blocks, s/he passed to a higher level of difficulty.

2 Supplementary Figures and Tables

2.1 Supplementary Figures

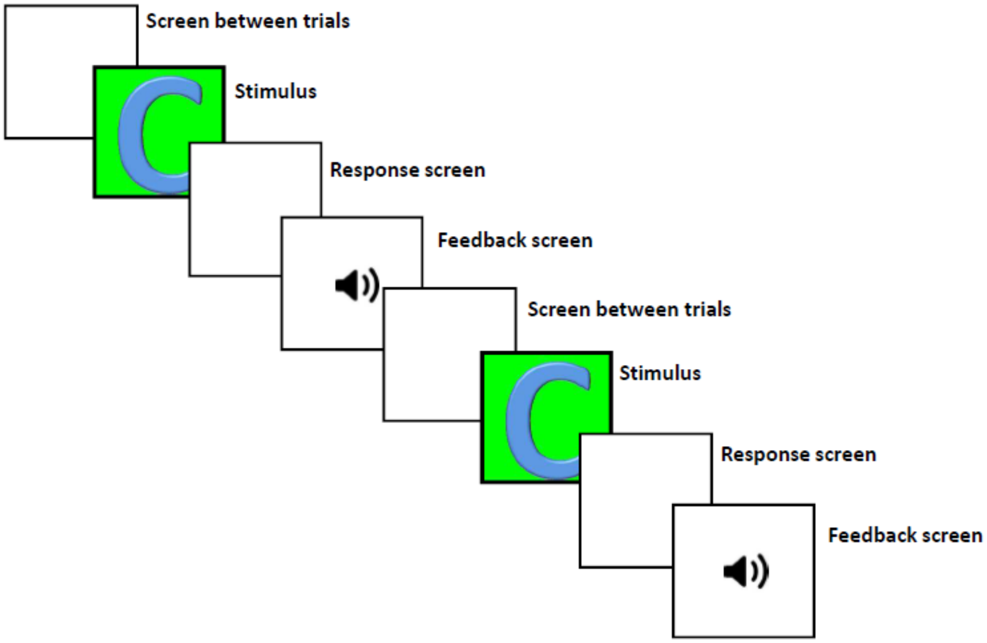

Supplementary Figure 1. Example of an *n*-back trial.

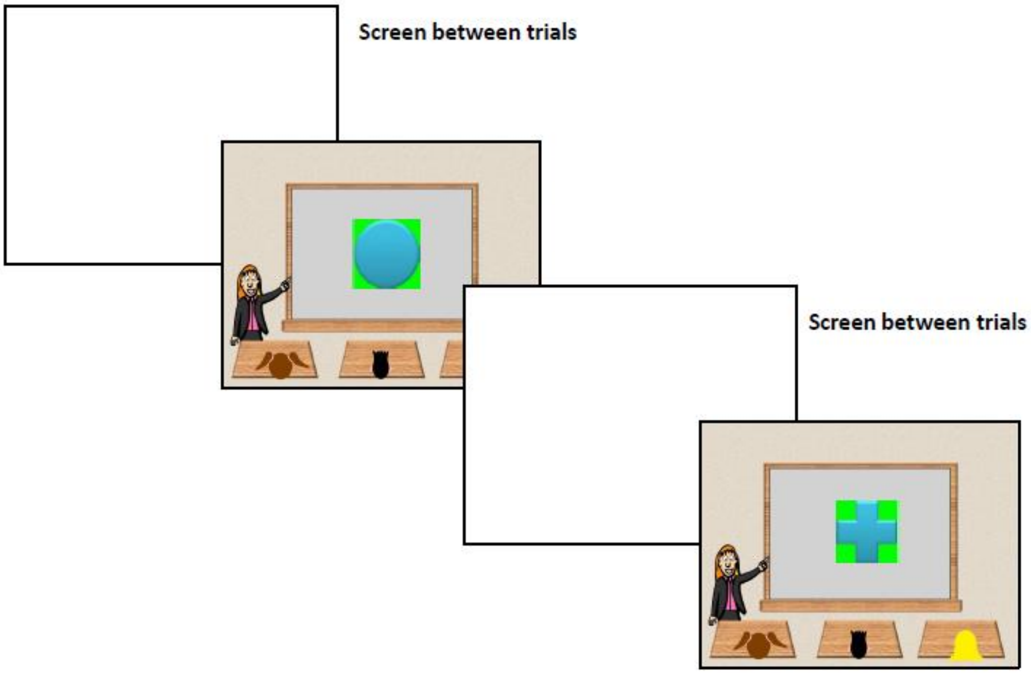

Supplementary Figure 2. Example of an abstract shapes trial.

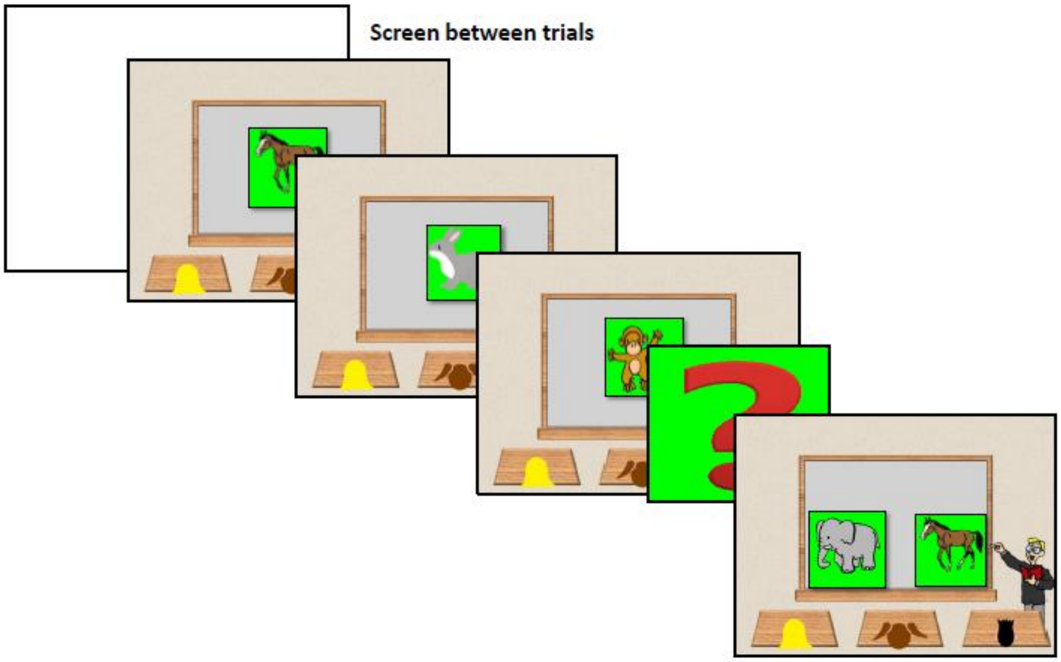

Supplementary Figure 3. Example of a working memory span trial.

2.2 Supplementary Tables

Supplementary Table 1. Math training tasks and descriptions used in this study.

Task	Description
**Rain of numbers**	In this activity the child was required to solve correctly the highest number of mathematical operations, within a time limit of two minutes. The mathematical operations presented were additions, subtractions, divisions, and multiplications.
**Numerical series**	This task consisted in to complete the highest number of numerical sequences, within a time limit of two minutes.
**Day-to-day situations**	This activity asked the child to solve the highest number of mathematical problems, within a time limit of two minutes.
**Magic numbers**	In this task the child was required to identify the highest number of digits within a matrix in a time limit of two minutes.
**Digits discrimination**	In this activity the child had to able to sort the highest number of digits list, in ascending order, and in decreasing order. At the same time, some digits were presented upside-down and the child had to identify the highest number of them as soon as possible.
**To complete operations**	The task presented to the child some mathematical operations without some numbers or without numeric symbols, and s/he was asked to complete the highest number of mathematical operations within a time limit of two minutes.

Supplementary Table 2. Adjusted R-squared and lower and upper confidence intervals for the multiple lineal regressions on children's cognitive skills improvements.

	Non-verbal IQ			% errors GNG type 1			% errors GNG type 2			% errors GNG type 3			% errors GNG total		
		95% CI			95% CI			95% CI			95% CI			95% CI	
	R^2_{adj}	Lower	Upper	R^2_{adj}	Lower	Upper	R^2_{adj}	Lower	Upper	R^2_{adj}	Lower	Upper	R^2_{adj}	Lower	Upper
Step 1	.30***			-.018			-.039			.015			-.003		
Course		-2.03	3.15		-3.16	6.54		-5.23	3.03		-4.47	3.71		-3.42	3.56
Gender		-7.16	3.67		-16.85	3.55		-11.60	5.68		-16.42	.75		-13.33	1.40
Pretraining scores		.30	.72		-.22	.28		-.17	.25		-.24	.20		-.19	.21
Step 2	.34***			-.016			.051			.28**			.17*		
WM Performance		.30	7.18		-6.57	3.76		-11.65	-.83		-13.43	-3.88		-10.75	-2.01
Math Performance		-4.19	1.35		-10.47	2.67		-5.34	3.12		-6.59	.89		-5.28	1.59

CI = Confidence Intervals; GNG = Go/NoGo task; * = $p < 0.05$; ** = $p < 0.01$; *** = $p < .001$.

Supplementary Table 3. Adjusted R-squared and lower and upper confidence intervals for the multiple lineal regressions on children's academic outcomes improvements.

	Math grades			Math Fluency			Reading abilities		
		95% CI			95% CI			95% CI	
	R^2_{adj}	Lower	Upper	R^2_{adj}	Lower	Upper	R^2_{adj}	Lower	Upper
Step 1	.64***			.66***			.73***		
Course		-.26	.07		-1.48	4.49		-.16	-.09
Gender		-.35	.29		-5.59	5.08		-.16	.30
Pretraining scores		.59	.97		.59	1.03		.76	1.12
Step 2	.63***			.64***			.75***		
WM Performance		-.26	.19		-3.07	4.60		-.01	.30
Math Performance		-.10	.26		-2.40	3.26		-.07	.17

CI = Confidence Intervals; GNG = Go/NoGo task; *** = $p < .001$.
